# Male Gender and Laparoscopic Cholecystectomy

**DOI:** 10.4103/1319-3767.49008

**Published:** 2009-04

**Authors:** Iqbal Saleem Mir

**Affiliations:** Department of Surgery, Govt. Medical College, Srinagar, Kashmir, India. E-mail: iqbalsurg@rediffmail.com

Sir,

I read with interest the paper on male gender and laparoscopic cholecystectomy by Al-Mulhim.[1] While the author needs to be commended for a thoroughly researched article, some points need to be clarified for the benefit of younger surgeons.

1. In the methodology, the author remarks that the pneumoperitoneum was created by closed method using a Veress needle. In some cases, where the patients had upper abdominal incisions, Hasson's technique was used. However, the author makes no comment about the patients who had undergone lower abdominal surgery especially by a lower midline incision. It is well known that omental and gut adhesions do develop underneath the scar, and sometimes these adhesions can be found covering a wider area internally than the skin incision. Faced with such a situation, it is wiser to use an open technique for the creation of pneumoperitoneum. Even though the gut injuries do occur with same frequency with open technique, in such cases the catastrophic vascular injuries are reduced.

Introduction of Veress needle at the Palmer's point in the left hypochondrium after aspirating the contents of stomach and ruling out splenic enlargement is another way to deal with such patients. Using optical trocars has not been proven to be immune to mishaps.

2. It is difficult to concur with the author on the irrelevance of preoperative ultrasound as a means to predict a difficult laparoscopic cholecystectomy. A routine ultrasound (USG) examination performed just before the admission can be helpful to the operating surgeon and can be done at the same time the patient is undergoing other preoperative investigations. A thick-walled gallbladder of more than 4 mm is a certain indicator about the possible difficult LC. In addition to this, the scan might document slipped stones in the common bile duct if the operator is experienced. This step becomes all the more essential if the USG has been performed at some other centre at an earlier date. The policy of preoperative USG examination should be adhered to by the surgeon till he masters the art of endosurgery and can tackle difficult cases with relative ease.

3. The idea of any publication is to present the work of the author to readers. In addition it stimulates other surgeons to look beyond their horizons. It is essential for the author to present a clear message to the readers whether the results can be reproduced by a surgeon at the start of his career. The author has to clearly demarcate the thin line in his article between calculated boldness and overzealous ventures. The reader on his part has to understand his limitations in surgical skills and stick to a step care approach especially during the early phase of the “learning curve.”
